# Threats of nursing productivity in the digital era: investigating the interplay between smartphones addiction and procrastination behavior among nurses

**DOI:** 10.1186/s12912-024-02218-y

**Published:** 2024-08-20

**Authors:** Ahmed Abdelwahab Ibrahim El-Sayed, Shimaa Fathy Daif Allah Goda, Gehan Galal Elbialy

**Affiliations:** 1 Nursing Administration Department, Faculty of Nursing, Alexandria University, 9 Edmond Vermont Street - Smouha, Alexandria, Egypt; 2 Faculty of Nursing, Alexandria University, 9 Edmond Vermont Street - Smouha, Alexandria, Egypt

**Keywords:** Digital, Productivity, Nursing, Threats, Smartphone, Addiction, Procrastination, Behavior, Nurses

## Abstract

**Background:**

Controlling smartphone addiction and procrastination among nurses is crucial for enhancing the productivity of both nursing and the healthcare system. Critical care nurses are highly vulnerable to smartphone addiction and procrastination behaviors than other groups. They may purposefully delay their tasks, a practice known as active procrastination, or inadvertently delay them, a practice known as passive procrastination.

**Aim:**

This study was designed to assess the prevalence of smartphone addiction and procrastination behavior among nurses, examine the effect of smartphone addiction on the active and passive procrastination behaviors, and explore the correlation between active and passive procrastination behaviors among nurses.

**Method:**

This is a descriptive correlational exploratory study that was conducted at 23 critical care units of one large educational hospital in Egypt. Data were collected from 360 nurses who were conveniently selected using three tools: the Smartphone Addiction Inventory, the New Active Procrastination Scale, and the Unintentional Procrastination Scale. Correlation and regression analyses were conducted to test the hypothetical relationship among the study variables.

**Results:**

This study revealed that 55.0%, 80.0%, and 45.3% of nurses had a moderate perceived level of smartphone addiction, active procrastination behavior, and passive procrastination behavior, respectively. There is a significant positive correlation between smartphone addiction and both nurses’ active and passive procrastination behaviors. Smartphone addiction accounts for 25% of the variance in nurses’ active procrastination behavior and 18% of the variance in their passive procrastination. Furthermore, there is a moderately significant negative correlation between nurses’ active procrastination behavior and their passive procrastination behavior.

**Conclusion:**

Nurses are exhibiting moderate levels of smartphone addiction and procrastination, which is a significant threat to the healthcare industry and nursing productivity. This requires technological, educational, and organizational interventions that foster active procrastination and combat passive procrastination behaviors among nurses.

**Implications:**

Continuous training programs are required to enhance time management skills among nurses and increase the awareness of nurse managers with the symptoms of smartphone addiction among nurses. Nurse leaders should early detect and address the addictive use of smartphones among nurses, identify potential procrastinators, and provide counseling to eradicate these behaviors in the workplace.

**Supplementary Information:**

The online version contains supplementary material available at 10.1186/s12912-024-02218-y.

## Introduction

Health systems all over the world have witnessed a remarkable and unprecedented reform due to smart technology proliferation and the smartphone revolution. The use of smartphones in recent contexts has played a significant role in shaping the professional and personal lives of health care providers, especially nurses [1]. Almost every nurse in this decade possesses a smartphone and uses it for a wide range of activities in nursing practice. A smartphone is a device that has both computing abilities and mobile communication technology [2].

Critical care nurses rely on smartphones for a myriad of tasks that enhance patient care and streamline their workflows. One of the primary uses is accessing electronic health records. Through smartphones, nurses can quickly retrieve comprehensive patient histories, lab results, and medication lists at the bedside [2]. This immediate access to vital information allows them to make informed decisions rapidly, which is crucial in critical care settings where time is often of the essence. Furthermore, updating patient records in real-time ensures accuracy and minimizes the risk of errors that could occur with manual entries or delayed documentation [3].

Effective communication is another significant task facilitated by smartphones. Critical care nurses use secure messaging apps to communicate swiftly with doctors, specialists, and other healthcare team members [4]. This capability is particularly valuable for coordinating care and seeking advice on patient management without the delays associated with pagers or in-person consultations. Additionally, smartphones enable video calls for remote consultations, allowing nurses to get immediate input from specialists who may not be on-site. This instant communication can lead to quicker interventions and better patient outcomes [1].

Smartphones also serve as essential tools for clinical decision support and education. Nurses can access a wide range of medical apps that provide drug interaction checkers, clinical guidelines, and diagnostic tools [3]. These resources help them make evidence-based decisions and ensure they are following the latest protocols. Furthermore, smartphones provide access to up-to-date research, continuing education modules, and training videos, allowing nurses to stay current with advancements in medical care [5]. On the other hand, abuse of smartphones to a level that can interfere with daily life may lead to smartphone addiction which is expressed as the excessive or compulsive use of smartphones that interferes with social relationships and hinders achieving personal life and work-related goals [6]. It is characterized by a lack of control over smartphone use with negative consequences and is considered a technological or behavioral addiction [5].

Smartphone addiction is an emergent issue of great concern among healthcare providers. In a meta-analysis of nineteen studies conducted worldwide, two out of five healthcare providers exhibited smartphone addiction [7]. Moreover, the prevalence of smartphone addiction among nursing staff is at the highest level comparable to other healthcare providers. In this respect, the meta-analysis of Osorio-Molina et al. (2021) revealed that 23% of nurses had a smartphone addiction comparable to that of 12% of physicians [8].

Lin et al. (2014) classified characteristics of smartphone addiction into four dimensions, namely compulsive behavior, functional impairment, withdrawal, and tolerance [9]. Compulsive behavior is the recurrent failure to resist the impulse to use the smartphone and is regarded as a central component of behavior addiction. Functional impairment is the influence of using a smartphone on time management and sleep. Withdrawal is the tendency to be impatient, irritable, and intolerable without a smartphone. Finally, tolerance is the tendency to spend more and more time using the smartphone [10].

Ma et al. (2021) highlighted that the addictive use of the smartphone may have detrimental effects on nurses’ physical, mental, and social health. Addictive smartphone use can produce anxiety, loneliness, insomnia, and somatization disorders [11]. Moreover, smartphone addicts reported having eye, neck, eating, and gastrointestinal disorders [12]. Additionally, obsessive-compulsive disorder behaviors, attention-deficit hyperactivity disorder, and reduced life satisfaction are frequent conditions among smartphone addicts [13]. Social life is also affected in terms of a loss of sense of community and depression. Furthermore, poor memory, lack of concentration, anxiety, and habitual procrastination are common findings among smartphone addicts [14].

Addictive use of smartphones is a dominant time waster as it makes nurses distracted by other irrelevant or tempting applications on the smartphone and information, which could progress to procrastination behavior [15]. Procrastination behavior is defined as the voluntary or involuntary delay of an intended task despite expecting to be worse off for the delay. It is characterized by symptoms such as difficulty initiating or completing tasks, lack of motivation or self-regulation, poor time management, avoidance of negative emotions or feedback, and rationalization of one’s behaviors [16]. Procrastination behavior can affect one’s work performance in ways such as missed deadlines, reduced quality of work, increased stress, and reduced satisfaction [17].

Active procrastination and passive procrastination are two distinct behavioral patterns that can be observed among nurses in the healthcare settings [18]. Active procrastination refers to the conscious choice to delay or postpone certain tasks, often with the intent of using the time pressure to enhance productivity and performance [19]. Nurses who engage in active procrastination may prioritize tasks based on perceived importance or deadlines, strategically allocating their time and resources to ensure critical responsibilities are addressed first. This approach can foster a sense of control and self-efficacy, as nurses feel they are making deliberate decisions about when and how to tackle their workload [20].

On the other hand, passive procrastination is characterized by a more subconscious and unintentional delay in completing tasks. Passive procrastinators do not intend to procrastinate; they often end up doing so due to an inability to make quick, effective decisions [21]. Affectively, an approaching deadline ultimately causes passive procrastinators to feel pressured, therefore creating pessimistic thoughts regarding their ability to achieve good results [22]. Also, they have trouble concentrating on the work at hand and find themselves spending excessive amounts of time on personal or non-work-related activities as a means of escaping difficult situations [7]. Nurses who procrastinate passively are frequently lethargic, reliant on others to assume accountability and finish tasks, and prone to time waste. They do not have a desire to manage the work environment with a high level of anxiety and self-regulation failure [20].

### Theoretical underpinnings of the study and hypotheses Formulation

This study was grounded in the Self-Regulation Theory (SRT) and the Dual-Process Theory of Self-Control. The SRT posits that individuals have a limited capacity for self-control, which can be depleted through various demands and distractions [23]. Smartphone addiction can be seen as a self-regulation failure, where individuals struggle to resist the temptation to engage with their smartphones, even when it interferes with their primary tasks and responsibilities. This depletion of self-control resources can then contribute to procrastination, as nurses may find it increasingly challenging to maintain focus and productivity in their work [20].

The Dual-Process Theory of Self-Control further expands on this concept by distinguishing between two types of procrastination: active and passive. Active procrastination involves a deliberate choice to delay a task due to perceived benefits In contrast, passive procrastination is characterized by the inability to resist distractions and the subsequent delay in task completion, which is more closely aligned with the detrimental effects of smartphone addiction [24]. Drawing on these theoretical frameworks, the proposed study aims to investigate how smartphone addiction, as a self-regulation challenge, may differentially impact active and passive procrastination behaviors among critical care nurses [25]. Based on the assumptions of both theories, the following hypotheses were formulated.

#### H_1_

Higher levels of smartphone addiction are associated with higher levels of active procrastination behavior among critical care nurses.

#### H_2_

Higher levels of smartphone addiction are associated with higher levels of passive procrastination behavior among critical care nurses.

#### H_3_

Nurses who are active procrastinators have a low tendency to procrastinate passively.

### Significance of the study and Research Gap

Smartphone distraction among critical care nurses poses a significant threat to patient safety. In an environment where every second counts, a nurse’s attention diverted to a smartphone can result in missed alarms, overlooked changes in patient conditions, and delayed responses to critical situations [20]. This distraction can lead to medication errors, such as administering the wrong dosage or medication, which can have severe or even fatal consequences [13]. Additionally, vital communications within the healthcare team may be compromised, leading to misunderstandings or incomplete handovers that leave critical information gaps. Such lapses in attention and communication can directly result in adverse events, jeopardizing the health and safety of patients in critical care [26].

Procrastination due to smartphone use further exacerbates these risks. When critical care nurses delay essential tasks like charting, monitoring patients, or addressing new symptoms due to smartphone-related procrastination, it can lead to significant delays in diagnosis and treatment [18]. This procrastination can cause a backlog of incomplete tasks, leading to rushed and potentially inadequate care as nurses attempt to catch up. Delayed interventions and treatments can worsen patient outcomes, particularly in a critical care setting where timely and precise actions are crucial. The combination of distraction and procrastination directly compromises patient safety, increasing the likelihood of adverse events and compromising the quality of care provided to the most vulnerable patients [19].

Understanding the connection between smartphone addiction and procrastination in this specialized setting is, therefore, of paramount importance [26]. Nurses working in critical care units often face demanding workloads, high-stress situations, and time-sensitive responsibilities. In such an environment, the temptation to engage in non-work-related smartphone activities, such as social media, messaging, or browsing, can be particularly strong. This phenomenon can lead to delayed task completion, increased errors, and compromised patient care [13].

Previous works stressed that critical care nurses are the most prominent procrastinators among nurses because of their prolonged use of smartphones in non-clinical activities, which is coincident with the work of Fiorinelli, et al. (2021), who found that critical care nurses are the most vulnerable group among health care providers for smartphone addiction [27]. Existing research has highlighted the potential negative consequences of smartphone addiction, including decreased productivity, reduced attention span, and impaired cognitive function. Investigating different forms of procrastination among nurses in healthcare settings has received little attention. The available studies addressed this issue in an academic context. For example, Meier (2022) found nursing students who use smartphones in an abusive pattern have a high tendency to procrastinate their academic work [28].

In the context of healthcare settings, previous studies addressed smartphone addiction among nurses at general care units [11] and intermediate care units [29], with no available studies conducted at critical care units. Additionally, the specific impact of smartphone addiction on procrastination behavior among critical care nurses remains largely unexplored. To address these research gaps, our study aims to delve into understanding the prevalence of different procrastination behaviors among critical care nurses. Also, our research is getting deeper into the issue of smartphone addiction, emphasizing its effect on the procrastination behavior of nurses.

Addressing smartphone addiction and procrastination among critical care nurses will significantly enhance their focus, efficiency, and overall job performance, leading to better patient care and outcomes. By reducing distractions and time wasted on non-essential activities, nurses can devote more attention to monitoring patients, administering timely treatments, and responding promptly to emergencies. This improved attentiveness and productivity directly contribute to higher quality care, reducing the likelihood of medical errors, and fostering a safer, more supportive environment for both patients and healthcare providers. Consequently, addressing these issues will not only improve nurses’ well-being and job satisfaction but also ensure optimal patient outcomes and enhanced overall healthcare delivery.

## Aim of the study

Objectives of this study are three-fold. First, this study aims to assess the prevalence of smartphone addiction and procrastination behavior among critical care nurses. Second, our study aims to explore the impact of smartphone addiction on nurses’ active and passive procrastination behaviors. Third, this study aims to examine the relationship between active procrastination and passive procrastination behaviors among nurses.

### Research questions

- What is the prevalence of smartphone addiction among critical care nurses?
- What is the prevalence of active and passive procrastination behaviors among critical care nurses?
- What is the effect of smartphone addiction on active and passive procrastination behaviors among nurses?
- What is the relationship between active procrastination and passive procrastination behavior among nurses?

## Method

### Research design

A descriptive-correlational exploratory research design was used to carry out this study. This design is used to describe or define a particular phenomenon and to test the feasibility of conducting a more extensive study [30].

### Setting and subjects

This study was conducted at Alexandria Main University Hospital, Egypt. It is the largest educational hospital with a bed capacity of more than 6760 and serves as a cornerstone for medical education, research, and patient care in Egypt. It provides non-paid health services to a wide scale of populations in lower Egypt. It is equipped with a wide variety of departments and units that cover almost all the common and rare medical and surgical specialties. This study was conducted at all Critical Care Units (CCUs) of this hospital (*N* = 23), which were divided into general critical care units (*n* = 7) and specialized intensive care units (*n* = 16).

The study sample included nurses who had at least six months of work experience, provide direct and indirect patient care, and agreed to participate in the study. Using G*power (version 3.1.9.7) for linear multiple regression: A sample size of 344 nurses was required to achieve a small effect size of 0.05 for F test, a power of 85%, a type 1 error of 0.05, with a total number of eight predictors. The sample size was increased to 360 to account for the potential incomplete, multiple, or extreme participants’ responses.

### Study tools

Data about nurses’ personal and work-related characteristics sheet was developed to collect data regarding age, gender, marital status, qualification, year of experience in the nursing profession and current working unit, previous attendance of workshops or courses about time management. In addition, three tools were used to collect data regarding the study variables:

#### Tool I: smart phone addiction inventory (SPAI)

This tool was developed by Lin et al. (2014) [9]. It was adopted by the researchers to measure to what extent nurses exhibit smartphone addiction. It consisted of 26 items, categorized into four dimensions: compulsive behaviors (9 items), functional impairment (8 items), withdrawal (6 items), and tolerance (3 items). Responses were obtained by using a 4-point Likert scale ranging from (1) = strongly disagree to (4) = strongly agree. Internal consistency was high; the overall Cronbach’s alpha score was 0.94 for the global scale [9]. The overall score of nurses’ smartphone addiction ranges from 26 to 104. The cut points of the scale were 26–51: low level, 52–77: moderate level, and 78–104: high level [9].

#### Tool II: new active procrastination scale (NAPS)

The New Active Procrastination Scales (NAPS) were developed by Chu & Choi (2005) as a 12-item scale to measure active procrastination behavior among nurses [31] and were later modified into a 16-item measure [32]. The modified version of NAPS was adopted to conduct this study. The NAPS focuses on four dimensions of active procrastination: a preference for pressure (4 items), an intentional decision to procrastinate (4 items), an ability to meet deadlines (4 items), and the ability to create a satisfactory outcome (4 items). The responses were obtained using a 7-point Likert scale ranging from not at all true (1) to very true (7). Internal consistency was high; the overall Cronbach’s alpha score was (α = 0.80) for the global scale. Scoring of all the items is in reversed form except items no. 9, 10, 11, and 12 [32]. The overall score of nurses’ active procrastination behavior ranges from 16 to 112. The cut points of the scale were 16–47: low level, 48–80: moderate level, and 81–112: high level [32].

#### Tool III: unintentional procrastination scale (UPS)

The Unintentional Procrastination Scale (UPS) was developed by Fernie et al. (2017) [21], and it was adopted to assess passive procrastination behavior among nurses. The UPS consists of six items that were measured using a 4-point Likert scale ranging from don’t agree (1) to very much agree (4), with a single-factor structure. Internal consistency was high; the overall Cronbach’s alpha score was.89 for the global scale [21].The overall score of nurses’ passive procrastination behavior ranges from 6 to 24. The cut points of the scale were 6–11: low level, 12–17: moderate level, and 18–24: high level [21].

### Tools translation, validity, and reliability

#### Tools translation

The tools were translated from English into Arabic to adapt to the Egyptian culture then back-to-back translation was done [33]. Two translators with a degree in translation and previous experience in written or oral translation in the field of healthcare and nursing were selected to translate the tool from English into Arabic. Each one was asked to translate the tool separately and independently. Both completed versions were unified, creating a single translated version of the questionnaires from English into Arabic. Then, two different bilingual translators with experience in healthcare and nursing related translation received the Arabic unified translated version and performed the back-translation of the tool from Arabic into English, resulting in a single back-translation. After both language versions became available, each item was checked to identify any inconsistencies and, wherever inconsistencies were found, the items were modified to stay as closely as possible to the original version in English. The final version of the questionnaires was revised by two members of the research team.

#### Content validity

The tools were tested for their face and content validity by five experts in the field of the study. They were three professors and two assistant professors from the nursing administration department, Faculty of Nursing, Alexandria University. The panel of experts highlighted errors in punctuation, typography, and word choice. Based on their suggestions, certain terms were changed, and the tools were put in their final configuration.

A pilot study was carried out on a number equal to 10% of nurses (*N* = 36) from Alexandria Main University hospital. It is intended to check and ensure the clarity of the tools, their applicability and feasibility, identify obstacles and problems that might be encountered during data collection, and establish the time required to complete the study questionnaire.

#### Construct validity

Both exploratory factor analysis (EFA) and confirmatory factor analysis (CFA) were used to assess the construct validity of the translated tools. To ensure the construct validity of the translated tools in the study context and among our population, we performed EFA to explore whether the factor structure remains consistent or differs in our sample. The EFA was applied using promax rotation with Kaiser-Meyer-Olkin (KMO) test to pinpoint the fundamental items that define dimensions of the corresponding tool. High values (close to 1.0) of KMO measure of sampling adequacy generally indicate that a factor analysis may be useful with data. If the value is less than 0.50, the results of the factor analysis probably won’t be very useful [34]. In our analysis, the KMO test of sample adequacy was 0.930, 0.920, and 0.879 for SPAI, NAPS, and UPS respectively, indicating the data were highly appropriate for factor analysis and show a high level of item-level common variance for each scale, see supplementary.

For SPAI, the EFA mirrored the scale’s four dimensions with the boldface loading ranged from 0.442 to 0.803, indicating that these items strongly contribute to their construct. With respect to NAPS, the EFA identified the four scale dimensions and all items had significant boldface loadings, ranging from 0.514 to 0.868, indicating a substantial factor contribution from the items. Regarding the UPS, the EFA showed a distinct and well-organized factor structure with high loadings for every item, ranging from 0.669 to 0.825, indicating a significant contribution from each item to the factor, see supplementary. The cut points of boldface loading were 0.32 (poor), 0.42 (fair), 0.55 (good), 0.63 (very good) or 0.71 (excellent) [35]. Based on these cut points, the results of EFA analysis revealed that the existing tools capture the same initially assumed dimensions, thus all items were retained.

We conducted the CFA using nine factors full Structural Equation Modeling (SEM); the SPAI (4 factors), NAPS (4 factors), and UPS (1 factor). The full model evaluates how well the factor structure of each tool suited the data. The models X^2^, Comparative Fit Index (CFI), Incremental Fit Index (IFI), and Root Mean Square Error of Approximation (RMSEA) were used to judge whether the model fit the data. The recommended X^2^ test should be insignificant; however, it may be affected by sample size. RMSEA of less than 0.05 is considered good, 0.05 to 0.08 is considered acceptable, 0.08 to 0.1 is considered marginal, and more than 0.1 is considered poor. The CFI and IFI values should be more than 0.9 [36]. The results of the CFA reveal a CFI = 0.1, and IFI = 0.1 indicating perfect fit, and the RMESA = 0.07 indicating acceptable fit, thus, we can conclude acceptable fit of the proposed model, see supplementary.

#### Reliability analysis

The reliability of the study tools was judged using the corrected item-total correlations and Cronbach’s alpha coefficient. To gauge how well each item matches the whole scale, a corrected the item-total correlations was employed. For the SPAI, it ranged from 0.642 to 0.869, reflecting that every item was positively and significantly correlated with the survey’s overall score. In respect to the NAPS, each item had a positive and significant relationship with the overall scale, as the corrected item-total correlations ranging from 0.814 to 0.854. According to the UPS’s corrected item-total correlations, which ranged from 0.692 to 0.811, each item was positively and strongly correlated with the scale overall score, see supplementary.

To assess the instruments’ internal consistency, Cronbach’s alpha was employed. SPAI’s subscales had Cronbach’s alpha values ranging from 0.825 to 0.844 for the subscales and 0.936 for the whole survey. The NAPS had Cronbach’s alpha values ranging from 0.810 to 0.887 and 0.928 for the total survey The UPS had a Cronbach’s alpha of 0.870. These findings demonstrate the three study tools had a strong internal consistency, see supplementary.

### Data collection

Data was collected from the staff nurses through hand-delivered questionnaires to study subjects in their work setting from different categories in the morning, evening, and night shifts. After an individualized interview with each nurse for about 10 min to explain the aim of the study, the needed instructions were given. They were given some general instructions regarding how to respond to questionnaires. Each subject took from 15 to 20 min to fill out the questionnaires. Each nurse was asked to return them to the researcher after the time required for each nurse to fill out three questionnaires. Data collection took two months, from the beginning of January 2024 to the end of February 2024.

### Statistical analysis

Data were analyzed using SPSS with version 25. All entered data were verified for any errors. Data were described using numbers, minimum, maximum, arithmetic mean, standard deviation. Categorical variables were described using frequency and percentage. The study employed inferential statistics to determine the correlation between the variables under investigation. The inter-correlation between the research variables was examined using Pearson’s coefficient (r). Correlation is considered perfect if r = of 1 or -1, strong if *r* <-0.5 or > 0.5, moderate if r between 0.3 and 0.5 or -0.5 and − 0.3, weak if *r* < 0.3 or > -0.3, and no association if *r* = 0. The significance of the correlation coefficient was judged at *p* ≤ 0.05. Furthermore, hierarchical regression analysis was performed to examine the extent of variation in nurses’ active and passive procrastination behaviors, as dependent variables, resulting from smartphone addiction while controlling for demographic variables.

## Results

Table 1 illustrates that 70.3% of nurses aged less than 30 years with a mean age of 28.29 ± 7.34., while 7.5% of them aged 40 to 59 years. Moreover, the highest percentage (63.1%) of nurses were female. According to level of education, more than two-thirds of the studied nurses (69.7%) had a bachelor’s degree in nursing, whereas the lowest percentage (1.9%) had a master’s degree in nursing. The same table reveals that more than three-quarters of nurses (77.5%) had from 1 to less than 10 years of experience in nursing, whereas the lowest percentage (7.2%) had 20 to 29 years of experience in nursing, with a mean of 7.84 ± 6.67 and a range < 1 to 36. As regards the previous attendance of workshops about time management skills, 96.7% of the studied nurses didn`t attend such workshops, whereas 3.3% attended such workshops in different settings where they answered it was helpful.

**Table 1:** Personal and work-related data of the study subjects (*N* = 360)

Demographic Data		No.	%
Age (years)	20-<30	253	70.3%
	30-<40	80	22.2%
	40–59	27	7.5%
	**Mean ± S.D.**	**28.29 ± 7.34**	
Gender	Males	133	36.9%
	Females	227	63.1%
Qualification	Diploma	24	6.7%
	Specialized diploma	78	21.7%
	Bachelor	251	69.7%
	Master	7	1.9%
Experience in nursing (year)	< 1 -<10	279	77.5%
	10-<20	55	15.3%
	20–29	26	7.2%
	**Mean ± S.D.**	**7.84 ± 6.67**	
experience in the current unit (year)	< 1	97	26.9%
	1-<10	228	63.3%
	10–29	35	9.7%
	**Mean ± S.D.**	**3.76 ± 4.64**	
Previous attendance of workshops about time management skills	Yes	12	3.3%
	No	248	96.7%
Degree of usefulness of workshop about time management skills	Useful	12	100%
	To some extent Not useful	0.0 0.0	0.0% 0.0%
Place of attendance	Educational setting	7	58.3%
	Health care setting	3	25%
	Other	2	16.7%
Previous attendance of courses about procrastination	Yes	4	1.1%
	No	356	98.9%
Degree of usefulness of courses about procrastination (*N* = 4)	Useful	4	100%
	To some extent Not useful	0.0 0.0	0.0% 0.0%
Place of attendance	Educational setting	2	50%
	Health care setting	1	25%
	Other	1	25%

Table 2 reveals the mean scores of the study variables. The mean score for smartphone addiction is 66.5 ± 16.3. Regarding dimensions of smartphone addiction, tolerance recorded the highest mean percent score (53.0% ± 28.5%), followed by functional impairment (52.6% ± 23.2%). On the other hand, compulsive behaviors recorded the lowest mean percent score (51.1% ± 22.5%), followed by withdrawal (51.9% ± 24.8%). Also, the mean score of the active procrastination behavior of nurses is 65.1 ± 12.2. Regarding dimensions of active procrastination behavior, the intentional decision to procrastinate recorded the highest mean percent score (58.6% ± 25.8%), followed by the ability to meet deadlines (52.8% ± 25.8%). On the other hand, the ability to create a satisfactory outcome recorded the lowest mean percent score (42.8% ± 28.7%), followed by preference for pressure (50.5% ± 25.3%). Also, the same table reveals that the mean score of unintentional procrastination behavior of nurses is 13.8 ± 4.7, with a mean percent score of 43.5% ± 25.9%.

**Table 2:** Mean scores of study variables

Scale / Dimensions	No. of Items	Mean ± S.D.	Mean %± S.D%
**Smartphone Addiction Inventory (SPAI)**	26	66.5 ± 16.3	52.0% ± 20.9%
Compulsive Behaviors	9	22.8 ± 6.1	51.1% ± 22.5%
Functional Impairment	8	20.6 ± 5.6	52.6% ± 23.2%
Withdrawal	6	15.3 ± 4.5	51.9% ± 24.8%
Tolerance	3	7.8 ± 2.6	53.0% ± 28.5%
**New Active Procrastination Scale (NAPS)**	**16**	**65.1 ± 12.2**	**51.2% ± 12.7%**
Preference for Pressure	4	16.1 ± 6.1	50.5% ± 25.3%
Intentional Decision to Procrastinate	4	18.1 ± 6.2	58.6% ± 25.8%
Ability to Meet Deadlines	4	16.7 ± 6.2	52.8% ± 25.8%
Ability to Create Satisfactory Outcome	4	14.3 ± 6.9	42.8% ± 28.7%
**Un-Intentional Procrastination Scale (UPS)**	**6**	**13.8 ± 4.7**	**43.5% ± 25.9%**

Table 3 reveals the correlation among study variables. Accordingly, there is a moderately significant positive correlation between smartphone addiction and both active and passive procrastination behavior (*r* = 0.533 *p* = 0.001, *r* = 0.468 *p* = 0.001), respectively. Moreover, there is a moderately significant negative correlation between active procrastination behavior and passive procrastination behavior among nurses (*r* = -0.582, *p* = 0.001).

**Table 3:** Correlation matrix between smartphone addiction, active and passive procrastination among nurses (*N* = 360)

		1	2	3	4	5	6	7	8	9	10	11
**1. Smartphone Addiction Inventory (SPAI)**	r											
	*p*											
**2. Compulsive Behaviors**	r	0.897										
	*p*	< 0.001*										
**3. Functional Impairment**	r	0.897	0.722									
	*p*	< 0.001*	< 0.001*									
**4. Withdrawal**	r	0.860	0.667	0.678								
	*p*	< 0.001*	< 0.001*	< 0.001*								
**5. Tolerance**	r	0.788	0.606	0.642	0.677							
	*p*	< 0.001*	< 0.001*	< 0.001*	< 0.001*							
**6. New Active Procrastination Scale (NAPS)**	r	0.533	0.408	0.455	0.512	0.539						
	*p*	< 0.001*	< 0.001*	< 0.001*	< 0.001*	< 0.001*						
**7. Preference for Pressure**	r	0.544	0.435	0.448	0.532	0.529	0.865					
	*p*	< 0.001*	< 0.001*	< 0.001*	< 0.001*	< 0.001*	< 0.001*					
**8. Intentional Decision to Procrastinate**	r	0.495	0.381	0.418	0.486	0.492	0.825	0.734				
	*p*	< 0.001*	< 0.001*	< 0.001*	< 0.001*	< 0.001*	< 0.001*	< 0.001*				
**9. Ability to Meet Deadlines**	r	-0.473	-0.385	-0.362	-0.491	-0.452	0.511	0.666*	0.577			
	*p*	< 0.001*	< 0.001*	< 0.001*	< 0.001*	< 0.001*	< 0.001*	< 0.001*	< 0.001*			
**10. Ability to Create Satisfactory Outcome**	r	0.443	0.343	0.360	0.443	0.452	0.724	0.589	0.434	0.698		
	*p*	< 0.001*	< 0.001*	< 0.001*	< 0.001*	< 0.001*	< 0.001*	< 0.001*	< 0.001*	< 0.001*		
**11. Un-Intentional Procrastination Scale (UPS)**	r	**0.468**	0.382	0.401	0.430	0.452	**-0.582**	0.532	0.392	-0.601	0.750	
	*p*	< 0.001*	< 0.001*	< 0.001*	< 0.001*	< 0.001*	< 0.001*	< 0.001*	< 0.001*	< 0.001*	< 0.001*	

r = Pearson correlation *Significant at *p* < 0.05

Table 4 portrays hierarchical multiple regression that examines the effect of smartphone addiction (overall) on active procrastination while controlling for socio-demographic characteristics among the studied nurses. Step 1 (Model 1): study the effect of sociodemographic data on active procrastination that illustrates the significant effect of sociodemographic data on active procrastination, where R2 = 0.068, which means that sociodemographic data account for 6% of the variance of active procrastination, with an overall significance level of F = 3.691, *p* = 0.001. Also, this model reveals a significant effect of total nursing experience on active procrastination (*p* = 0.003), which means that total nursing experience is a predictor of active procrastination. Step 2 (Model 2): this model, after adding smartphone addiction as an independent variable, examined the effect of smartphone addiction and socio-demographic data on active procrastination. This model illustrates the significant effect of smartphone addiction on active procrastination (*p* < 0.001 and R2 = 0.319), which means that both socio-demographic data and smartphone addiction together account for 31% of the variance of active procrastination. On the other hand, the effect of smartphone addiction alone on active procrastination is R2 change = 0.251, which means that smartphone addiction accounts for 25% of the variance in active procrastination.

**Table 4:** Hierarchical multiple regression demonstrating the effect of smartphone addiction on active procrastination among studied nurses (*n* = 360)

Variable	Step 1 (Model 1)					Step 2 (Model 2)				
	B	S.E.	Β	t	*P*	B	S.E.	Β	T	*p*
(Constant)	55.389	5.833		9.496	0.000	35.133	5.300		6.628	0.000
Age	0.057	0.273	0.033	0.209	0.835	-0.060	0.234	-0.035	-0.257	0.798
Gender (1 = Male)	-0.542	1.434	0.021	0.378	0.706	1.323	1.229	0.050	1.077	0.282
Educational Level	-0.783	1.241	-0.039	-0.631	0.529	0.492	1.069	0.024	0.460	0.646
Nursing Experience at Current Place	0.648	0.296	0.237	2.190	0.029	0.767	0.254	0.280	3.024	0.003
Total Nursing Experience	-0.891	0.296	-0.468	-3.011	0.003	-0.611	0.254	-0.321	-2.403	0.017
Previous attendance of workshop about use of smartphone	-2.374	3.642	-0.034	-0.652	0.515	-2.176	3.117	-0.031	-0.698	0.486
Previous attendance of workshop about use of Procrastination	0.961	6.271	0.008	0.153	0.878	-2.335	5.376	-0.019	-0.434	0.664
Smartphone Addiction Inventory (SPAI)						0.313	0.028	0.516	11.377	0.000
	R = 0.261 (F = 3.691, *p* = 0.001)					R = 0.565 (F = 20.587, *p* < 0.001)				
	R²=0.068					R²=0.319 R² Change = 0.251 (F = 129.435, *p* < 0.001)				

Table 5 portrays hierarchical multiple regression that examines the effect of smartphone addiction (overall) on passive procrastination behavior while controlling for socio-demographic characteristics among the studied nurses. Step 1 (Model 1): study effect of socio-demographic data on passive procrastination that illustrates significant effect of socio-demographic data on passive procrastination, R2 = 0.090, which means that sociodemographic data account for 9% of variance of passive procrastination, with an overall significance level of F = 4.998, *p* < 0.001). Also, this model reveals a significant effect of total nursing experience on passive procrastination (*p* = 0.000), which means that total nursing experience is a predictor of passive procrastination. While Step 2 (Model 2), after adding smartphone addiction as an independent variable, studied the effect of smartphone addiction and socio-demographic data on passive procrastination. Also, it reveals a significant effect of smartphone addiction on passive procrastination (*p* < 0.001 and R2 = 0.270), which means that both socio-demographic data and smartphone addiction together account for 27% of the variance of passive procrastination. On the other hand, the effect of smartphone addiction alone on passive procrastination is R2 change = 0.180, which means that smartphone addiction accounts for 18% of the variance in passive procrastination.

**Table 5:** Hierarchical multiple regression demonstrating the effect of smartphone addiction on passive procrastination among studied nurses (*n* = 360)

Variable	Step 1 (Model 1)					Step 2 (Model 2)				
	B	S.E.	Β	t	*p*	B	S.E.	Β	t	*P*
(Constant)	40.948	11.771		3.479	0.001	5.880	11.206		0.525	0.600
Age	1.069	0.551	0.303	1.941	0.053	0.867	0.494	0.245	1.753	0.080
Gender (1 = Male)	-3.769	2.894	-0.070	-1.302	0.194	-2.416	2.599	-0.045	-0.930	0.353
Educational Level	-1.694	2.505	-0.041	-0.676	0.499	0.513	2.259	0.012	0.227	0.821
Nursing Experience at Current Place	0.965	0.597	0.173	1.615	0.107	1.170	0.536	0.209	2.183	0.030
Total Nursing Experience	-2.615	0.597	-0.672	-4.379	0.000	-2.131	0.538	-0.548	-3.961	0.000
Attending Sessions about Smartphone Effective Use	-5.437	7.349	-0.038	-0.740	0.460	-5.094	6.590	-0.035	-0.773	0.440
Attending Sessions about Procrastination	6.561	12.656	0.027	0.518	0.604	0.855	11.365	0.003	0.075	0.940
Smartphone Addiction Inventory (SPAI)						0.542	0.058	0.437	9.317	0.000
	R = 0.301 (F = 4.998, *p* < 0.001)					R = 0.520 (F = 16.289, *p* < 0.001)				
	R²=0.090					R²=0.270 R² Change = 0.180 (F = 86.800, *p* < 0.001)				

## Discussion

This study aims to assess the prevalence of procrastination behavior among nurses and its relationship with smartphone addiction. This study goes beyond classical passive procrastination behavior and sheds light on the active form of procrastination among nurses. The findings revealed that this behavior is moderately prevalent among nurses. In addition, smartphone addiction is a powerful predictor to both active and passive procrastination behavior among nurses.

### Smartphone addiction among nurses

Controlling smartphone addiction is one of the success issues toward productive and sustainable nursing practice. Unfortunately, the current study found that more than half of nurses had a moderate level of smartphone addiction. This suggests that this problem is not isolated or limited to a small subset of nurses, but rather a more widespread challenge faced by the profession. This may be attributed to the studied nurses working in critical care units that have unique dynamics that create a relentless and fast-paced atmosphere, often leading to feelings of overwhelming pressure and exhaustion among critical care nurses. High workloads and work-related stress are inevitable phenomena among critical care nurses. These issues make critical care nurses use their smartphones in an abusive manner as a way to cope with numerous stressors in their clinical practice. Also, factors such as the need to stay connected, fear of missing out on important information, and the allure of social media platforms may contribute to the moderate level of smartphone addiction in this study.

These reasons are supported by the results of Fiorinelli et al. (2021), who revealed a direct correlation between a high level of smartphone addiction and a high perceived work overload [27]. In this context, Ma et al. (2021) [11], Karataş et al. (2022) [37], Zhong et al. (2022) [38], and Liu et al. (2023) [1] reported that healthcare professionals who were experiencing work-related stress and burnout were more likely to have a smartphone addiction. One of the key implications of this finding is the potential distraction caused by smartphone use among nurses. With constant notifications and easy access to social media platforms, nurses may be tempted to check their phones frequently, compromising their attention during critical tasks. This behavior not only reduces productivity but also poses safety risks for patients. It is crucial to address this issue by raising awareness and promoting healthy smartphone habits to maintain the well-being of both nurses and patients.

Additionally, this finding emphasizes the need for policies and guidelines regarding smartphone use in healthcare settings. Healthcare facilities should consider implementing educational programs or workshops to raise awareness about the risks of excessive smartphone use. Furthermore, the study suggests that the nature of nursing work itself may contribute to smartphone addiction. Nurses often face high levels of stress and long working hours, making them more likely to turn to their smartphones for escapism or entertainment. These factors should be considered when developing interventions to address smartphone addiction among nurses, as they highlight the need for effective stress management strategies and work-life balance initiatives.

This finding is the case in the studies of Piscotty et al. (2016) [39], Celikkalp et al. (2020) [40], Karataş et al. (2021) [37], Ma et al. (2021) [11], and Noghan et al. (2023) [29]. These studies found a considerable prevalence of smartphone addiction among nurses. In this context, Osorio-Molina et al. (2021) illustrated the highest level of smartphone addiction among nursing staff, comparable to other healthcare providers [8]. The study by Di Muzio et al. (2019) found that nurses had a high level of smartphone addiction that negatively affected their work performance and increased the risk of medical errors [41]. Moreover, the work of Savci et al. (2021) [42], Mohamed et al. (2020) [43] and Abdelaliem et al. (2023) [44] revealed a moderate to high level of smartphone addiction among nursing students. Moreover, studies conducted on medical students by Farooqui et al. (2018) [45], Sethuraman et al. (2018) [46], and Lei et al. (2020) [47] revealed a moderate level of smartphone addiction among medical students.

Likewise, studies conducted on various employees by Ai et al. (2021) revealed that the majority of the private higher education staff had moderate levels of smartphone addiction [48]. Additionally, Derks et al. (2021) assessed this phenomenon among employees from various occupations and revealed that a considerable percent of participants reported interruptions from private smartphone use [4]. Also, Derakhshanrad et al. found that office workers experienced a moderate to high level of smartphone addiction [49]. On the other hand, smartphone addiction is reported at a low level among nurses in the studies of Liu et al. (2023) [1], McBride et al. (2013) [50], and Planitz et al. (2013) [51]. This may be attributed to a study conducted in a different context than a critical unit, and this context is marked by decreased workload and low stress and conflict levels.

### Procrastination behavior among nurses

This study found that active and passive procrastination behaviors are prominent since the highest percentage of nurses had moderate levels of both active and passive procrastination behaviors. These levels in the current study are expected due to several contributing factors. These factors could be classified into personal factors and work-related factors. Personal factors include the dominance of females in this study, who had a high tendency to procrastinate due to multiple role overload. This is supported by the results of the hierarchical regression model, which revealed gender is an active predictor of procrastination behavior, where being male is associated with a low tendency to procrastinate, unlike females. This finding is consistent with Ahmad & Hussain’s (2020) declaration that female employees were more inclined to procrastinate than males [52].

Moreover, qualification is another personal factor since a considerable percentage of participants had a diploma with an associated degree where there was no curriculum regarding procrastination taught to them and time management in their curricula received little attention. In addition, there is a negative relationship between qualification and procrastination behavior, with a high tendency to procrastinate visible among low-qualification nurses, meaning that low-qualification nurses have a high tendency to procrastinate. As regards age, the majority of nurses had less than 30 years of experience. Many factors among this group make them procrastinators, like easy distraction, low self-regulation, an increase in task aversion, a lack of time management abilities, less decisiveness, high anxiety, and not having certain talents [19].

Years of experience is another personal factor that contributed to the high level of procrastination behavior in the current study, since a considerable percentage of participants had 1 to less than 10 years of experience. This study found there is a negative relationship between nursing experience and procrastination behavior. This means that a limited number of years of experience is associated with a high tendency to procrastinate. This may be attributed to those with limited years of experience who do not have enough time to receive training related to time management.

As related to workplace-related factors that contribute to a high level of procrastination. Nurses working at this critical unit have a heavy workload, mandatory overtime, a lack of recognition for their contributions, bad workplace incentives, a stressful work environment, professional role ambiguity, a lack of interest and desire, and burnout. These reasons are supported by the results of Hen et al. (2018), who revealed that professional role ambiguity and situational determinants were the primary reasons for procrastination [53]. In this context, Hutmanová et al. (2022) [54] and Johansson et al. (2023) [55] revealed that a stressful work environment and a lack of interest and desire are the main reasons for procrastination. Moreover, Ma et al. (2021) found that work procrastination is associated with burnout.

This finding is the case in the studies of Basirimoghadam et al. (2020) [56], Babaie et al. (2022) [19], and Moghadam et al. (2019) [57]. These studies found a considerable prevalence of procrastination behavior among nurses. Moreover, the work of Zeng et al. (2024) revealed a moderate level of procrastination behavior among nursing and medical students [58]. On the other hand, Rezaei et al. (2016) revealed that nearly three-quarters of the staff nurses had low procrastination [59]. This may be explained by cultural differences, good hospital services, and available resources that facilitate task performance.

### Smartphone addiction and procrastination behavior among nurses

This study found that smartphone addiction is a key factor leading to the cultivation of procrastination behavior among nurses since results revealed a positive correlation between smartphone addiction and active procrastination and passive procrastination. This is supported by the results of the hierarchical regression model, since smartphone addiction accounts for 25% of the variance in active procrastination and 18% of the variance in passive procrastination.

This relationship suggests that the ubiquity of smartphones in modern life is exacerbating the problem. Nurses may find themselves increasingly drawn to the constant stimulation and distractions offered by their smartphones, leading them to prioritize non-essential tasks over their core responsibilities. This tendency can create a vicious cycle, where smartphone use fuels procrastination, which in turn drives further smartphone use as a coping mechanism. This gives the impression that smartphone addiction is a powerful predictor of procrastination behavior. Subsequently, this implied that nurse leaders and decision-makers must assess the issue of smartphone addiction and abusive use of smartphones to apply strategies to control them and build a sustainable, productive work environment free from procrastination.

The result of this study is in parallel with Ma et al. (2021) [11], and Savci et al. (2021) [42] who revealed that procrastination behavior is predominant among habitual users of smartphones. In this context, a study conducted during the COVID-19 pandemic found that smartphone addiction directly influenced procrastination in young adults [60].On the other hand, a study done by Tárrega-Piquer et al. (2023) revealed that there is no relationship between the tendency to procrastinate and levels of smartphone addiction [61]. This contradiction could be attributed to the fact that procrastination is a multifaceted phenomenon influenced by various factors. Nurses’ procrastination behavior cannot be solely attributed to smartphone addiction; instead, it results from a combination of individual traits, work-related stress, and environmental factors.

### Linkage between active and Passive Procrastination Behavior among nurses

This study delved into the fascinating topic of procrastination among nurses and unearthed an intriguing finding—a negative correlation between active procrastination and passive procrastination. This result challenges commonly held beliefs about procrastination and sheds light on unique behavioral patterns prevalent within the nursing profession. The study revealed that nurses who reported higher levels of active procrastination also exhibited lower levels of passive procrastination. This suggests that nurses who actively delay tasks tend to compensate by engaging in other constructive activities rather than wasting time. These nurses seemingly possess the ability to prioritize and manage their time effectively, utilizing it to their advantage. Furthermore, the negative correlation uncovered in the study can be seen as a positive phenomenon from a productivity standpoint. Nurses who engage in active procrastination may experience improved work-life balance, enhanced decision-making skills, increased levels of satisfaction, and able to tackle tasks efficiently when necessary.

This result highlights the complexity of this phenomenon and the importance of nuanced understanding. Simply categorizing all procrastination as detrimental may oversimplify the issue and lead to ineffective interventions. Healthcare leaders and policymakers must recognize that procrastination exists on a spectrum, with active procrastination potentially serving as an adaptive coping mechanism in high-stress environments. By acknowledging this distinction, they can work to create work environments and support systems that foster the productive aspects of active procrastination while addressing the more problematic passive procrastination behaviors. This balanced approach can help nurses better manage their workloads and maintain optimal patient care without succumbing to the negative consequences of procrastination.

This result is supported by Chu and Choi (2005) [31], Choi & Moran (2009) [32], Fernie et al. (2017) [21], and Wessel et al. (2019) [22], who found that the majority of active procrastinators had positive outcomes and achieved their objectives, which is contrary to passive procrastinators. Moreover, Onuegbu (2020) [62], and Sanecka (2022) [18] in their works declared that active procrastination results in positive, satisfactory outcomes whereas passive procrastination is characterized as a dysfunctional type of task delay. Moreover, Aziz et al. (2019) [63], and Zohar (2019) [64] clarified that correlations between the big five personality traits are more visible among passive procrastinators than active ones, which supports why the relationship among them goes negative.

## Implications of the study and future directions

This study has broader implications for the nursing profession and healthcare organizations. Firstly, the widespread nature of smartphone addiction and procrastination behavior among nurses underscores the need for a comprehensive and targeted approach to address it. Healthcare organizations must recognize the scope of the problem and allocate resources and strategies to tackle it effectively. Secondly, healthcare organizations must take proactive steps to mitigate the impact of smartphone addiction on nurses’ work performance. This may involve implementing policies and protocols that limit smartphone usage during work hours, providing education and training on the risks of excessive smartphone use, and offering support and resources to help nurses develop healthier coping strategies for managing stress and workload.

Thirdly, reducing procrastination behavior among nurses requires interventions that consider the different manifestations of this behavior. Strategies to address active procrastination may need to focus on time management skills, workload prioritization, and the promotion of a work culture that recognizes the potential benefits of strategic delay. Conversely, interventions targeting passive procrastination would likely need to address deeper-seated issues such as poor self-regulation, a lack of motivation, or underlying mental health concerns. Tailoring approaches to the specific type of procrastination behavior exhibited is a promising strategy that helps healthcare agencies develop more effective solutions to support nurses and mitigate the negative consequences of this issue.

Future research could be directed to assess procrastination behavior in other contexts like oncology units, emergency departments, and operating rooms, compare the prevalence of this behavior among different healthcare providers, and develop scales that could be used by nurses to self-assess their level of this behavior. Also, future studies could aim to assess the effect of psychological therapies on the symptoms of smartphone addiction among nurses and develop novel measures to prevent smartphone addiction among nurses using artificial intelligence apps that detect prolonged use and misuse.

## Conclusion

The current study sheds light on smartphone addiction and procrastination behavior among nurses. It is evident that the future of nursing productivity and quality of nursing care is at high risk since procrastination behavior and smartphone addiction are moderately visible among nurses, which necessitates urgent measures to be taken to buffer the negative effects of both issues. Also, smartphone addiction is a serious workplace issue that could significantly reinforce procrastination behavior among nurses. Addressing these issues requires a multifaceted approach that combines technological, educational, and organizational interventions. Such interventions should target fostering active procrastination and combating passive procrastination behavior among nurses.

### Electronic supplementary material

Below is the link to the electronic supplementary material.

Supplementary Material 1

## Data Availability

The datasets used and/or analysed during the current study are available from the corresponding author on reasonable request.

## Abbreviations

SPAI: Smartphone Addiction Inventory
NAPS: New Active Procrastination Scale
UPS: Unintentional Procrastination Scale
CFA: Confirmatory Factor Analysis
EFA: Exploratory Factor Analysis
