# Supplementary material for: Threats of nursing productivity in the digital era: investigating the interplay between smartphones addiction and procrastination behavior among nurses
Source: BMC Nurs. 2024 Aug 20;23:577. doi: 10.1186/s12912-024-02218-y (PMC11337763; doi:10.1186/s12912-024-02218-y)
Supplement: Supplementary file 1 — Supplementary Material 1 [file 12912_2024_2218_MOESM1_ESM.pdf]

Study Tools Validity and Reliability

Table (1): Exploratory factor analysis and factor loadings (EFA) :Smart Phone Addiction Inventory (SPAI)

After rotation Promax with Kaiser Normalization						
**Item number**	**Sections**	**Factor 1**	**Factor 2**	**Factor 3**	**Factor 4**	**Communalities**
item1	Part 1	0.691				0.491
item2	Part 1	0.581				0.658
item3	Part 1	0.463				0.594
item4	Part 1	0.751				0.566
item5	Part 1	0.733				0.571
item6	Part 1	0.669				0.611
item7	Part 1	0.544				0.501
item8	Part 1	0.473				0.305
item9	Part 1	0.658				0.553
item10	Part 2		0.687			0.539
item11	Part 2		0.701			0.615
item12	Part 2		0.740			0.567
item13	Part 2		0.764			0.612
item14	Part 2		0.519			0.351
item15	Part 2		0.615			0.433
item16	Part 2		0.667			0.548
item17	Part 2		0.509			0.612
item18	Part 3			0.559		0.550
item19	Part 3			0.520		0.608
item20	Part 3			0.725		0.568
item21	Part 3			0.433		0.474
item22	Part 3			0.442		0.651
item23	Part 3			0.438		0.584
item24	Part 4				0.803	0.681
item25	Part 4				0.703	0.523
item26	Part 4				0.722	0.610
**Kaiser-Meyer-Olkin Measure of Sampling Adequacy = 0.930**						

The boldface indicates salient (> 0.30) loading

Table (2): Exploratory factor analysis and factor loadings (EFA) Tool II: New Active Procrastination Scale (APS)

After rotation Promax with Kaiser Normalization						
**Item number**	**Sections**	**Factor 1**	**Factor 2**	**Factor 3**	**Factor 4**	**Communalities**
item1	Part 1	0.838				0.633
item2	Part 1	0.841				0.726
item3	Part 1	0.865				0.687
item4	Part 1	0.851				0.736
item5	Part 2		0.514			0.599
item6	Part 2		0.868			0.785
item7	Part 2		0.845			0.789
item8	Part 2		0.778			0.685
item9	Part 3			0.632		0.542
item10	Part 3			0.795		0.769
item11	Part 3			0.716		0.754
item12	Part 3			0.545		0.753
item13	Part 4				0.642	0.712
item14	Part 4				0.728	0.716
item15	Part 4				0.777	0.753
item16	Part 4				0.784	0.785
**Kaiser-Meyer-Olkin Measure of Sampling Adequacy = 0.920**						

The boldface indicates salient (> 0.30) loading

Table (3): Exploratory factor analysis and factor loadings (EFA) Tool III: Unintentional Procrastination scale (UPS)

Principal Component Analysis.			
**Item number**	**Sections**	**Factor 1**	**Communalities**
item1	Part 1	0.669	0.448
item2	Part 1	0.756	0.571
item3	Part 1	0.825	0.681
item4	Part 1	0.811	0.657
item5	Part 1	0.797	0.635
item6	Part 1	0.813	0.661
**Kaiser-Meyer-Olkin Measure of Sampling Adequacy = 0.879**			

The boldface indicates salient (> 0.30) loading

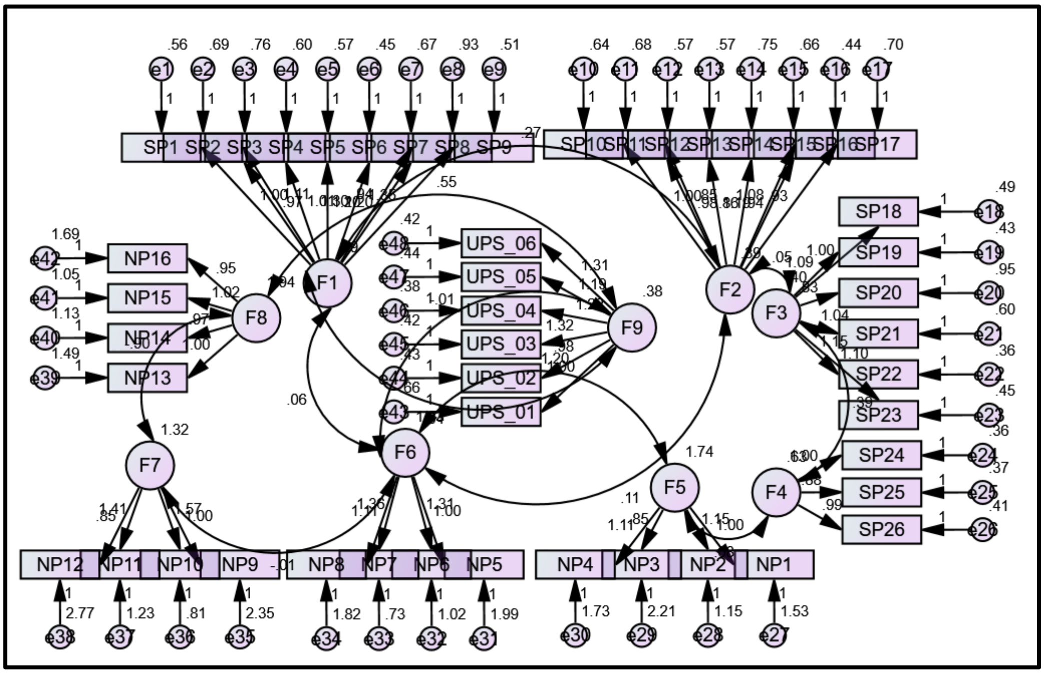

Figure (1): Confirmatory factor analysis (CFA) by Structure Equation Modeling (SEM)

Model fit parameters CFI; IFI; RMSEA (1.000; 1.000; 0.07).
CFI = Comparative fit index; IFI = incremental fit index; and RMSEA = Root Mean Square Error of Approximation.
Model χ^2; significance 9.964 (0.001*)

F1: Compulsive behaviors
F2: Functional impairment
F3: Withdrawal
F4: Tolerance
F5: A preference for pressure
F6: Intentional decision to procrastinate
F7: An ability to meet deadlines
F8: The ability to create a satisfactory outcome
F9: Unintentional Procrastination scale (UPS)

Table (4): Corrected Item-Total Correlations for Tool I: Smart Phone Addiction Inventory (SPAI)

Item number	Sections	Correlation
item1	Part 1	0.642*
item2	Part 1	0.703*
item3	Part 1	0.647*
item4	Part 1	0.634*
item5	Part 1	0.685*
item6	Part 1	0.737*
item7	Part 1	0.680*
item8	Part 1	0.550*
item9	Part 1	0.733*
item10	Part 2	0.680*
item11	Part 2	0.650*
item12	Part 2	0.705*
item13	Part 2	0.769*
item14	Part 2	0.620*
item15	Part 2	0.659*
item16	Part 2	0.746*
item17	Part 2	0.629*
item18	Part 3	0.724*
item19	Part 3	0.774*
item20	Part 3	0.638*
item21	Part 3	0.739*
item22	Part 3	0.817*
item23	Part 3	0.761*
item24	Part 4	0.862*
item25	Part 4	0.851*
item26	Part 4	0.869*

Correlation: Pearson coefficient

*: Statistically significant at $p \leq 0.05$

Table (5): Corrected Item-Total Correlations for Tool II: New Active Procrastination Scale (APS)

Item number	Sections	Correlation
item1	Part 1	0.814*
item2	Part 1	0.860*
item3	Part 1	0.744*
item4	Part 1	0.793*
item5	Part 2	0.765*
item6	Part 2	0.857*
item7	Part 2	0.873*
item8	Part 2	0.822*
item9	Part 3	0.759*
item10	Part 3	0.853*
item11	Part 3	0.844*
item12	Part 3	0.704*
item13	Part 4	0.859*
item14	Part 4	0.869*
item15	Part 4	0.877*
item16	Part 4	0.854*

Correlation: Pearson coefficient
*: Statistically significant at $p \leq 0.05$

Table (6): Corrected Item-Total Correlations for Tool III: Unintentional Procrastination scale (UPS)

Item number	Sections	Correlation
item1	Part 1	0.692*
item2	Part 1	0.749*
item3	Part 1	0.825*
item4	Part 1	0.803*
item5	Part 1	0.791*
item6	Part 1	0.811*

Correlation: Pearson coefficient
*: Statistically significant at $p \leq 0.05$

Table (7): Cronbach's Alpha for Tool I: Smart Phone Addiction Inventory (SPAI)

Item number	Sections	Cronbach's Alpha if Item Deleted
item1	Part 1	0.830
item2	Part 1	0.823
item3	Part 1	0.830
item4	Part 1	0.831
item5	Part 1	0.825
item6	Part 1	0.818
item7	Part 1	0.826
item8	Part 1	0.840
item9	Part 1	0.819
**Cronbach's for Part 1**	**0.844**	
item10	Part 2	0.817
item11	Part 2	0.821
item12	Part 2	0.812
item13	Part 2	0.802
item14	Part 2	0.827
item15	Part 2	0.820
item16	Part 2	0.806
item17	Part 2	0.825
**Cronbach's for Part 2**	**0.836**	
item18	Part 3	0.809
item19	Part 3	0.796
item20	Part 3	0.832
item21	Part 3	0.807
item22	Part 3	0.784
item23	Part 3	0.800
**Cronbach's for Part 3**	**0.834**	
item24	Part 4	0.761
item25	Part 4	0.761
item26	Part 4	0.752
**Cronbach's for Part 4**	**0.825**	
**Cronbach's for overall**	**0.936**	

Table (8): Cronbach's Alpha for Tool II: New Active Procrastination Scale (APS)

Item number	Sections	Cronbach's Alpha if Item Deleted
item1	Part 1	0.756
item2	Part 1	0.721
item3	Part 1	0.809
item4	Part 1	0.782
**Cronbach's for Part 1**	**0.815**	
Item5	Part 2	0.840
Item6	Part 2	0.787
Item7	Part 2	0.774
Item8	Part 2	0.815
**Cronbach's for Part 2**	**0.848**	
Item9	Part 3	0.772
Item10	Part 3	0.700
Item11	Part 3	0.706
Item12	Part 3	0.810
**Cronbach's for Part 3**	**0.810**	
Item13	Part 4	0.860
Item14	Part 4	0.850
Item15	Part 4	0.846
item216	Part 4	0.863
**Cronbach's for Part 4**	**0.887**	
**Cronbach's for overall**	**0.928**	

Table (9): Cronbach's Alpha for Tool III: Unintentional Procrastination scale (UPS)

Item number	Sections	Cronbach's Alpha if Item Deleted
item1	Part 1	0.869
item2	Part 1	0.852
item3	Part 1	0.837
item4	Part 1	0.841
item5	Part 1	0.845
item6	Part 1	0.840
**Cronbach's for overall**	**0.870**	
